# Draft Genome Sequence of Klebsiella sp. Strain C31 Isolated from a Malaysian Tropical Peat Swamp Forest

**DOI:** 10.1128/genomeA.00560-18

**Published:** 2018-06-21

**Authors:** Chin Chin Too, Kuan Shion Ong, Markus J. Ankenbrand, Sui Mae Lee, Catherine M. Yule, Alexander Keller

**Affiliations:** a School of Science, Monash University Malaysia, Petaling Jaya, Malaysia; b Tropical Medicine and Biology Multidisciplinary Platform, Monash University Malaysia, Petaling Jaya, Malaysia; c Department of Bioinformatics, Biocenter, University of Würzburg, Würzburg, Germany; d Center for Computational and Theoretical Biology, University of Würzburg, Würzburg, Germany

## Abstract

We report here the draft genome of Klebsiella sp. strain C31, a bacterial isolate from the North Selangor peat swamp forest in Malaysia. The putative genes for the biogeochemical processes of the genome were annotated and investigated.

## GENOME ANNOUNCEMENT

The genus Klebsiella includes pathogenic bacteria found ubiquitously in soil, surface waters, plants, and humans (1). The species K. pneumoniae has been reported with the ability of nitrogen fixation in nature (2, 3). We isolated Klebsiella sp. strain C31 from a peat sample taken at a depth of 90 cm in the North Selangor Peat Swamp Forest, Malaysia, and sequenced the whole genome of the isolate to investigate its potential roles in carbon and nitrogen cycles in extreme habitats.

Klebsiella sp. strain C31 was enriched on primary enrichment medium (4) and then cultured on minimal medium, using glucose as the carbon source (5), at Monash University Malaysia. DNA extraction was performed using the pure culture incubated in tryptic soy broth for 36 h at 30°C, following the protocol of the QIAamp DNA Mini Kit (Qiagen, Hilden, Germany). Library preparation was conducted following the protocol of the Nextera XT DNA library preparation kit (Illumina, San Diego, CA, USA), followed by whole-genome sequencing on the Illumina NextSeq 500 platform (150-bp paired-end reads) at the Biocenter, University of Würzburg, Germany. DNA raw reads were corrected and assembled *de novo* using SPAdes version 3.10.1 (6). Gene annotation was implemented with two tools, BlastKOALA (7) and Prokka version 1.12 (8). The annotated genes were then mapped onto pathways available in the KEGG database (9).

The genome contained 41 contigs with a total length of 5,265,977 bp (*N*_50_ = 306,003 bp) and a GC content of 57.53%. There were 4,860 coding DNA sequences (CDSs), 1 transfer-messenger RNA (tmRNA), 9 rRNAs, and 87 tRNAs observed. Klebsiella sp. strain C31 possessed genes that encode the enzymes methanol dehydrogenase and formate dehydrogenase, indicating its potential ability to utilize methanol and formate, with CO_2_ as the end product. It also possessed genes associated with various hydrolases such as alpha-amylase, cellulase, chitinase, alpha-glucosidase, beta-glucosidase, alpha-galactosidase, beta-galactosidase, and sucrase, which catalyze the hydrolysis of cellulose and hemicellulose from plant cell walls and various carbohydrates.

This bacterial strain was predicted to perform dissimilatory nitrate reduction, as the genes *nar*G, *nar*H, and *nar*I, as well as *nir*B and *nir*D, were detected. Further investigation of the genome will provide more information on the potential roles this strain plays in tropical peat swamp forest ecosystems.

### Accession number(s).

This whole-genome shotgun project has been deposited at DDBJ/ENA/GenBank under the accession number QBUZ00000000. The version described in this paper is version QBUZ01000000.
